# Attempt to Untangle the Prion-Like Misfolding Mechanism for Neurodegenerative Diseases

**DOI:** 10.3390/ijms19103081

**Published:** 2018-10-09

**Authors:** Daniela Sarnataro

**Affiliations:** Department of Molecular Medicine and Medical Biotechnology, University of Naples Federico II, School of Medicine, Via S. Pansini 5, 80131 Naples, Italy; daniela.sarnataro1@gmail.com; Tel.: +39-0817-464-557

**Keywords:** Aβ, aggregation, amyloid, APP, misfolding, prion protein, prion-like, PrP, seeds, tau

## Abstract

The misfolding and aggregation of proteins is the neuropathological hallmark for numerous diseases including Alzheimer’s disease, Parkinson’s disease, and prion diseases. It is believed that misfolded and abnormal β-sheets forms of wild-type proteins are the vectors of these diseases by acting as seeds for the aggregation of endogenous proteins. Cellular prion protein (PrP^C^) is a glycosyl-phosphatidyl-inositol (GPI) anchored glycoprotein that is able to misfold to a pathogenic isoform PrP^Sc^, the causative agent of prion diseases which present as sporadic, dominantly inherited and transmissible infectious disorders. Increasing evidence highlights the importance of prion-like seeding as a mechanism for pathological spread in Alzheimer’s disease and Tauopathy, as well as other neurodegenerative disorders. Here, we report the latest findings on the mechanisms controlling protein folding, focusing on the ER (Endoplasmic Reticulum) quality control of GPI-anchored proteins and describe the “prion-like” properties of amyloid-β and tau assemblies. Furthermore, we highlight the importance of pathogenic assemblies interaction with protein and lipid membrane components and their implications in both prion and Alzheimer’s diseases

## 1. Introduction

Prion diseases are incurable neurological disorders that produce a broad range of symptoms in mammalian species including humans (Creutzfeldt–Jacob Disease (CJD), Gerstmann–Sträussler–Scheinker (GSS), Fatal Familial Insomnia (FFI), Kuru) and cattle (Bovine Spongiform Encephalopathy (BSE)).

Prion diseases are characterized by the misfolding of a normal protein (cellular prion protein, PrP^C^) into the pathological β-sheet-rich isoform defined scrapie prion protein (PrP^Sc^), which represents an essential component in the pathophysiology of neurodegenerative prion diseases whose etiology can be infectious, sporadic or genetic.

In the case of infectious prion diseases, the formation of nascent prions has been proposed to be driven by a direct interaction between the pathogenic PrP^Sc^ template and the endogenous PrP^C^ substrate [1]. By contrast, in the genetic forms, alteration in PrP^C^ conformation may be induced by a genetic mutation in *Prnp* gene encoding PrP^C^ [1,2].

PrP^C^ is a glycosylphosphatidylinositol (GPI)-anchored protein located on the cell surface in lipid–enriched microdomains also called lipid rafts [3,4]. Interestingly, contrasting data indicate that (i) the lipid and protein environment at the plasma membrane might be favourable for PrP^C^–PrP^Sc^ interaction and conversion [5] or that (ii) they can have a protective role in pathological scrapie conversion of PrP mutants [6]. These findings highlight the critical and controversial role of lipid rafts in protein misfolding [5,7].

Some mutations leading to genetic prion diseases, characterized by PrP^Sc^ accumulation, are not only present in the C-terminal domain of PrP^C^ but are also present in the GPI-attachment signal, implying that the GPI-anchor signal itself can also play a role in neurodegeneration [8]. The GPI-anchor remodelling steps through the passage to the ER and Golgi (critical cellular organelles for chaperoning folding processes) are essential for the final protein localization in the lipid rafts at the outer leaflet of the plasma membrane, which in turn together with endosomal recycling compartment, has been considered to participate in PrP^Sc^ conversion [9].

It has recently emerged the concept of propagating misfolding by which the normal protein, PrP^C^, becomes misfolded and gain-of-function mechanisms associated with this misfolding not only propagate further PrP^C^ misfolding in neighboring cells, but can also infect other organisms.

The ability of protein particles, deriving from misfolding and aggregation of amyloid-β (Aβ), tau, α-synuclein (α-syn), superoxide dismutase 1 (SOD1), to transfer from one cell to another, similar to misfolded PrP, accounts for the widespread pathophysiology seen in neurodegenerative disorders such as Alzheimer’s disease (AD), Parkinson’s disease (PD), amyotrophic lateral sclerosis (ALS) and Huntington’s (HD) disease [10].

Thus, the concept of propagating misfolding, together with the emerging demonstrations of “cell non-autonomous” mechanism of intercellular transfer of protein inclusions [11,12], represents the basics for “prion-like” disorders definition of AD, PD, ALS and HD.

Here, we review the latest findings on the mechanisms controlling protein folding/misfolding focusing on the peculiarity of GPI-anchored proteins ER quality control, with special attention to PrP^C^, and analyse the “prion-like” properties of amyloid-β and tau assemblies. Finally, we highlight the importance of misfolded/pathogenic assemblies’ interaction with membrane components and their roles in the pathogenesis of both prion and Alzheimer’s diseases.

## 2. ER/Golgi Quality Control and the Role of GPI-Anchor in Protein Conformation

### 2.1. Quality Control of PrP: The ERAD Pathway

The mammalian PrP^C^ is a secretory glycoprotein, whose signal peptide located at the N-terminal domain, targets the synthesis and regulates the import into the endoplasmic reticulum [7]. The N-terminus is rather unstructured and mediates copper internalization. PrP^C^ also has a GPI signal peptide at the C-terminus, which regulates the attachment of the GPI anchor. The well-structured C-terminus containing α-helices, can mediate, together with the N-terminus, the ER import of PrP^C^.

Before reaching the plasma membrane, PrP^C^ is subjected to a quality control process, which operates to ensure its correct folding [13].

It has been estimated that about 10% of total PrP^C^ is misfolded when synthesized [13], hence the quality control system is extremely important in the cell.

**Figure 1:**
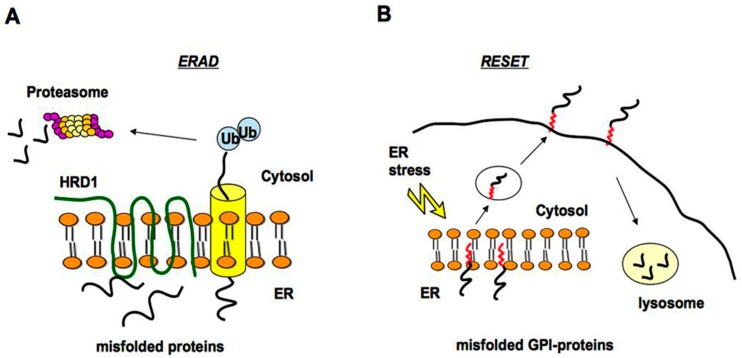
The ERAD and RESET pathway for degradation of misfolded proteins. (**A**) Misfolded proteins accumulated in the ER are retrotranslocated from the ER to the cytosol through the translocon (yellow). They are then polyubiquitinated and degraded by the proteasome system. HRD1 (an E3 ubiquitin-ligase) is localized into the ER membrane and mediates the transfer of ubiquitin from ubiquitin-conjugated enzyme E2 to substrates; (**B**) the ER-stress induced pathway called RESET regulates the degradation of misfolded GPI-anchored proteins, which dissociate from the resident ER chaperones (not illustrated) leaving the ER and reaching the cell surface transiently before lysosomal degradation.

The ER and Golgi apparatus have a crucial role in the quality control of secreted and membrane proteins [14]. Indeed, misfolded proteins are retained at the ER and those that are not able to properly fold are degraded via the ERAD (ER-associated degradation pathway) by the proteasome system after cytosolic ubiquitination [15,16]. Degradation of misfolded ER luminal and membrane proteins is promoted by an HRD1 (HMG-CoA Reductase Degradation 1 Homolog) complex, which represents a high conserved ERAD machinery in the ER (Figure 1A) [17]. Different pathological mutants of the GPI-anchored PrP^C^ have been shown to be degraded by the ERAD and may accumulate in the cytosol [18,19]. Furthermore, the impairment of the ubiquitin-proteasome system by aggregation prone proteins is associated with prion-induced neurodegeneration [20].

### 2.2. Quality Control of PrP: The RESET Pathway

Because of the involvement of GPI-APs (GPI-anchored proteins) in neurodegenerative prion diseases [7,9], the quality control of GPI-APs has been extensively studied.

Although various misfolded GPI-APs accumulate after proteasome inhibitor treatment, suggesting that the ERAD is involved in their turnover [6,13,21,22], it has been reported that misfolded GPI-anchored proteins do not pass through the canonical ERAD pathway but seem to be targeted to the alternative ER stress-induced pathway called RESET [23]. Thanks to RESET, misfolded GPI-APs dissociate from resident ER chaperones, leaving the ER and accessing the cell surface transiently before degradation in lysosomes (Figure 1B). The primary mechanism of ER egress of misfolded GPI-APs depends on the ER export receptor Tmp21, which may act as a chaperone ensuring safe trafficking through the secretory pathway to lysosomes [23].

The GPI anchor, after attachment to a protein, is subjected to a series of remodeling steps on both the sugar and lipid moieties, which impact the functions of the anchor itself, in the intracellular trafficking and membrane dynamics [24]. The fact that GPI-APs result in being refractory to ERAD pathway degradation [25] may be due to the topologic problem for the ERAD machinery, posed by the covalently attached lipid in the luminal leaflet of the bilayer. Nonetheless, at present, the mechanisms that allow the cell to discriminate properly folded from misfolded GPI-APs at the plasma membrane are still unknown.

Interestingly, if on one hand we have previously demonstrated that misfolded PrP mutants lacking the GPI anchor (PrPΔGPI) are not localized at the plasma membrane but mainly released in cell culture media [26], on the other hand, they could be efficiently routed to the ERAD [27]. In addition, the same PrP mutant (H187R), with a functional GPI anchor is degraded in lysosomes [25]. These observations together with the recent RESET pathway description, led to the postulation that the presence of a GPI anchor might obstruct ERAD for sterical hindrance [23].

However, findings from Sikorska and colleagues [28] demonstrated that the misfolded GPI-anchored protein Gas1* in yeast is targeted to HRD1-dependent ERAD pathway, ruling out that a GPI anchor obstructs ERAD. Instead, the normally decreased ERAD for Gas1* is caused by remodelling of its GPI anchor (which occurs in all GPI-APs), thus proposing the new concept that the canonical remodelling of GPI anchor universally limits the ER quality control of GPI–APs.

More recent studies [8] demonstrated that misfolded GPI-anchored PrP (PrPΔ214–229, partially lacking the C-terminal domain) is directed to the secretory pathway and under steady-state it follows the RESET pathway. Interestingly, under stress conditions or aging, it reaches the Golgi apparatus where it remained, inducing the activation of neurotoxic signalling with concomitant activation of p38-MAPK, without activation of UPR (Unfolded Protein Response) pathways [29].

These findings reveal the role of Golgi apparatus in the quality control of protein folding [30]. Indeed, Golgi quality control can route misfolded proteins back to the ER or forward to lysosomes for degradation [31], for which the state of oligomerization/aggregation [32], rather than exact folding state of monomeric protein, can act as regulator principle for Golgi quality control system. However, the role of Golgi apparatus in prion disease pathogenesis is far from being clarified.

In prion-infected mice, Uchiyama et al. [33] found a lower expression of PrP^C^ and other GPI-anchored proteins at the neuronal plasma membrane, while these proteins accumulated in the Golgi apparatus, finding that prions disturb the post-Golgi trafficking of membrane proteins. It seems that molecular mechanisms involving Rab GDP dissociation inhibitor α (GDI), regulating Rabs function in vesicular trafficking, represent a key factor in the role of Golgi apparatus stress response. Indeed, it has been reported that the PrP mutants accumulating in the Golgi apparatus are able to upregulate GDI, whose silencing rescues post-Golgi transport [33]. If p38-MAPK activation may represent a mechanistic aspect of Golgi stress still has to be determined.

### 2.3. Quality Control of APP

In response to ER stress due to A23187 calcium ionophore, APP (Amyloid Precursor Protein) is rapidly degraded by the ubiquitin-proteasome system (UPS) [34]. Thus, it is conceivable to hypothesize that chronic ER stress and deregulation of UPS contribute to AD progression.

A direct involvement of the ubiquitin-ligase HRD1 in APP ubiquitination and degradation was revealed by the finding of HRD1 interaction with misfolded APP in neuronal cells, and by experiments where suppression of HRD1 expression caused APP accumulation and generation of Aβ associated to ER stress [35,36].

The γ-secretase/BACE1 amyloidogenic processing of APP give rise to the terminal fragment C99/CTFβ that can be subsequently cleaved by γ-secretase to produce Aβ. The amount of C99/CTFβ is considered determinant for AD, as the availability of C99 as substrate for γ-secretase increases the probability of γ-secretase cleavage and Aβ production. The C99 fragment has been found degraded by the ubiquitin-proteasome pathway [37], via polyubiquitination of its cytosolic lysine residues, and inhibition of the proteasome shifted its degradation to the acidic lysosomal pathway. These findings highlighted the crosstalk between ERAD and lysosomes to avoid protein accumulation and toxicity.

### 2.4. Quality Control of Tau

The microtubules-associated tau protein (MAPT) is able to associate with proteins of the ER and ER-associated degradation pathway, rendering this degradation route dysfunctional [38]. Indeed, association of tau with ER proteins (such as ribosomal proteins L28 e P0) was different between control and AD brains, tau/P0 association being more robust in AD, thus suggesting possible pathogenic processes by which tau leads to cellular dysfunction, such as ribosomal dysfunction, which has been associated with the pathogenesis of AD [39].

Importantly, the participation of the protein quality control network in the regulation of tau aggregates’ formation and propagation has been reported by very recent findings, where the heat shock protein Hsp70 chaperone is able to block the early stages of tau aggregation by suppressing tau nuclei formation [40]. Specifically, Hsp70 sequesters tau fibrils into protective complex neutralizing propagation of tau seeds and, consequently, the toxic properties of soluble tau oligomers towards lipid membrane, whose integrity is known to be impaired by tau oligomers [41].

## 3. “Prion-Like” Misfolding of Aβ and Tau: Implication for Alzheimer’s Disease

Protein misfolding and aggregation constitute a hallmark for many neurodegenerative diseases, including AD. It has been reported that the key molecular event in the pathogenesis of prion diseases, is the conformational conversion of PrP^C^ into PrP^Sc^ by a not yet understood process in which PrP^Sc^ binds to PrP^C^ promoting its pathological conversion.

A growing body of research supports the concept that misfolding and aggregation of the endogenous protein fragment amyloid-β initiates and sustains the pathogenesis of AD, which is characterized by the presence of Aβ plaques and neurofibrillary tangles (NFTs), these latter consisting of intracellular bundles of hyperphosphorylated tau protein [42,43].

**Figure 2:**
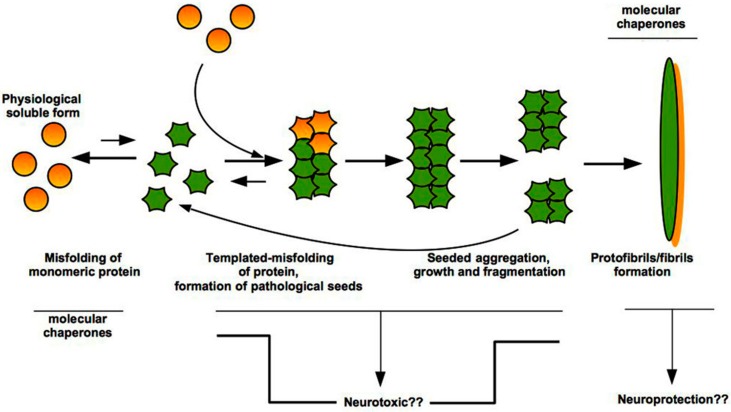
Prion-like mechanism of misfolded protein aggregation. Misfolding of normal physiological form of a protein and formation of pathological seeds is a rare and energetically unfavourable event, based upon exposition of amide groups and high concentration of a given protein. Genetic mutations or environmental factors (e.g., exposure to infective PrPSc, pesticides) can induce the conversion from soluble normal form to insoluble pathological oligomers and larger species that aggregate and fibrillize. Once a seed has formed, thanks to a template-assisted misfolding, each single molecule can acquire a different shape and add to growing aggregates. These latter can be fragmented generating new seeds that are able to accelerate the aggregation, giving life to fibrils formation. Question marks (??) indicate open issues.

Both Aβ and tau, as well as PrP^C^, assume a tertiary structure (or fold) rich in β-sheets which in turn promotes the self-assembly of monomers into small oligomeric species, with neurotoxic properties, and fibrillary assemblies (Figure 2) [44,45].

The small size and hydrophobicity of the oligomers are able to induce cellular dysfunctions [46].

Molecular chaperones have been shown to interact with both oligomers that represent the small species at the beginning of the aggregation events, and with the amyloid fibrils that are the end-product of this process [46]. The action of the chaperones is aimed not only at interfering with amyloid formation, but also at inhibiting directly the toxic nature of the aberrant species. In particular, chaperones increase the size of oligomers and mask the hydrophobic moieties on their surface.

Moreover, the effect of chaperone-mediated stabilization of fibrils represents a protective strategy for the cell because the resulting stabilized inclusions are unable to release cytotoxic oligomers, which are the basis to promote secondary nucleation events [47].

### Mutations and Polymorphisms That Promote Aβ Misfolding and Aggregation Increase the Risk of AD

The Aβ deposition and NFTs, once formed, affect large areas of the brain with the progression of AD, which is typified by neuronal loss and neuroinflammation [48]. Aβ seeds, like PrP prions, can reach the brain from outside the CNS (Central Nervous System) [49]. In addition, unlike inclusions made of tau or scrapie-prion protein, β-amyloid deposits form in the extracellular space [11].

Enhancement of Aβ release from the APP or its tendency to self aggregate have been reported to be regulated by genetic mutations in APP, which can cause autosomal dominant and recessive AD [50,51]. In contrast, a rare mutation in an APP gene (APPA673T) reduces both the production of Aβ and its tendency to aggregate [52]. All together these findings point to the critical and controversial role played by Aβ in the pathogenesis of AD.

Evidence for the prion-like seeding of Aβ in humans comes from recent findings that describe a significant Aβ plaque accumulation in four out of eight hormone recipients in Great Britain who died of CJD [53]. It was discovered that some batches of pituitary growth hormone (GH), derived from a subset of patients with iatrogenic CJD, were contaminated by PrP prions [54]. The accumulation of Aβ plaques in the brain of these patients raises the possibility that some batches were Aβ contaminated. Because the GH recipients were died of CJD, it remains unknown whether they would have developed AD. Because CJD patients, who received dura mater prion-contaminated transplants, were found to have increased Aβ plaques [55], another possibility is that prions increase Aβ levels through the deviation of PrP^C^ signalling (associated to p38 and JNK-stress kinases), which could impair Aβ clearance via a decreased activity of MMP-9 metalloprotease [56].

Tauopathies are pathologically and phenotypically diverse and include AD, corticobasal degeneration, chronic traumatic encephalopathy and frontotemporal dementia (FTDP-17) [57,58,59,60]. Tau is expressed in neurons and at lower levels in glia [57] and the main attributed function is to stabilize microtubules [61]. Differential splicing results in the production of tau isoforms with either three (3R) or four (4R) repeats, and one or two N-terminal inserts (reviewed in [62]). Intriguingly, certain intronic and some exonic mutations can alter the 3R/4R ratio increasing unbound tau and leading to aggregation [57,58,59,60], which by mounting evidence is thought to occur in a prion-like manner [63].

In AD, both the three- and the four-repeats tau make up the neurofibrillary lesions (reviewed in [11]). Moreover, filaments produced in vitro, starting from the microtubule-binding domain of 4R human tau, were endocytosed by the cells inducing filament formation by full length tau following a direct contact through a prion-like mechanism [64]. This observation prompted the authors to postulate that tau aggregates can propagate a misfolded state to the inside of the cells.

However, within the proposed mechanism of tau conformational-templating and seeding, the aggregation of tau monomer to tau seeds must first take place. A recent study from Strang et al. [62] indicates that specific regions within tau play a key role in regulating aggregation and seeding.

In particular, P301L and S320F tau mutants are uniquely able to aggregate with seeding when compared to WT tau, and aggregated P301L tau failed to sequester soluble WT tau into insoluble aggregates in the HEK293 cells seeding model [62]. Moreover, because of a lower tendency of P301L tau to aggregate in the presence of WT tau expression, it is possible that WT tau may reduce template conformation of P301L tau. Concerning S320F tau, cryo-electron microscopy studies indicate that the mutation would strongly stabilize tau amyloid fibrils fold and subsequent fibril polymerization [65].

Stereotypical spatial and temporal spreading of tau inclusions has been noted in different tauophaties [66,67]. The finding of inclusions made of different tau isoforms in different diseases is consistent with the existence of tau strains, similar to the prion strains made of different PrP^Sc^ conformers.

Tau is the most commonly misfolded proteins in human neurodegenerative diseases, which besides AD include some cases of GSS [68], where intraneuronal tau inclusions coexist with extracellular Aβ deposits and prion protein, respectively. However, there are other tauopathies, such as Pick’s disease and FTDP, which are characterized by tau inclusions in the absence of extracellular deposits (reviewed in [11]).

## 4. The Intrinsically Disordered Proteins: A Common Feature of Proteins Associated with Misfolding Diseases

Aggregation and amyloidogenesis constitute the consequences of protein misfolding, which leads to neurodegenerative diseases. Various intrinsically disordered proteins (IDPs) are involved in pathogenesis of these disorders [69].

IDPs are biologically active proteins without stable tertiary structure, characterized by the presence of disorder-promoting residues in their amino acid compositions [70]. PrP^C^ and Shadoo (this latter being a member of the prion protein family) are characterized by extended unstructured/disordered domains (IDD) and, together with APP and tau, they well represent the class of IDPs. A unique property of IDPs is their conformational plasticity and incompleteness of “folding code” [71]. In other words, they don’t have any confirmed/ordered 3D conformation [72] and possess the ability to adopt very different structures in the bound state while interacting with diverse partners [70]. IDPs can exist in a wide variety of conformational forms, and protein aggregation is the ultimate outcome of abnormal IDP regulation in the cell.

An interesting analysis made by Das and colleagues [73] revealed that more than 80% of human proteins in the disordered protein database (DisProt + IDEAL) contained one or more amyloidogenic region (AR), generally positioned in the internal part of a protein sequence, and that the sequences in the ARs showed a mixed conformational adaptability towards α-helix, β-sheet and coil conformation. The amyloidogenic region often acts as a nucleation center and governs protein aggregation that eventually leads to formation of β-sheet rich amyloid fibers.

Nevertheless, besides the AR ability to direct protein aggregation, the C-terminal domain of certain PrP mutants linked to inherited human prion diseases, was described to be necessary to drive the nascent protein to the ER. Indeed, PrP mutants lacking the C-terminus were inefficiently imported to the ER forming neurotoxic cytosolic conformers [74].

**Figure 3:**
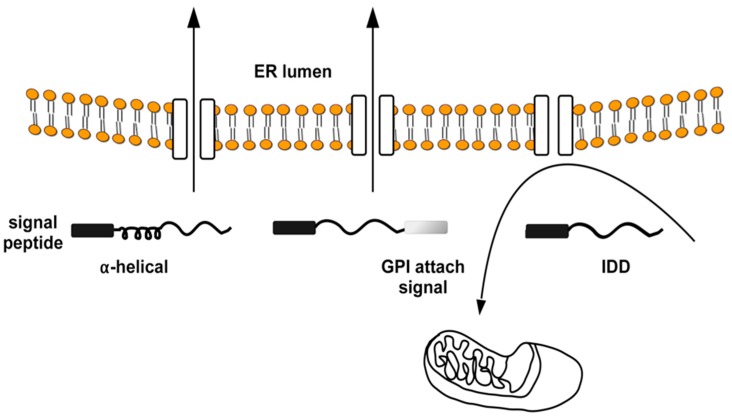
Intrinsically disordered proteins and ER import. The ER signal peptides (black rectangle) of the prion protein-like protein Shadoo and APP can mediate alternative targeting to mitochondria. Structural elements (e.g., α-helical domain, GPI-attachment signal) within the nascent polypeptide chain can determine the targeting direction (towards the ER or mitochondria) of these signal peptides. The ER import of nascent chains is efficiently promoted by each signal peptide if the peptide contains α-helical domains, while it targets unstructured polypeptides (IDD, intrinsically disordered domain) to mitochondria. Arrows indicate the direction of protein traffic across the ER.

However, impaired ER import of IDPs is a general phenomenon and not a specific feature of PrP mutants. This observation derives from studies where it was shown that IDPs require alpha-helical domains in addition to the N-terminal signal peptide for efficient Sec61-mediated transport into the ER (Figure 3) [74,75].

**Figure 4:**
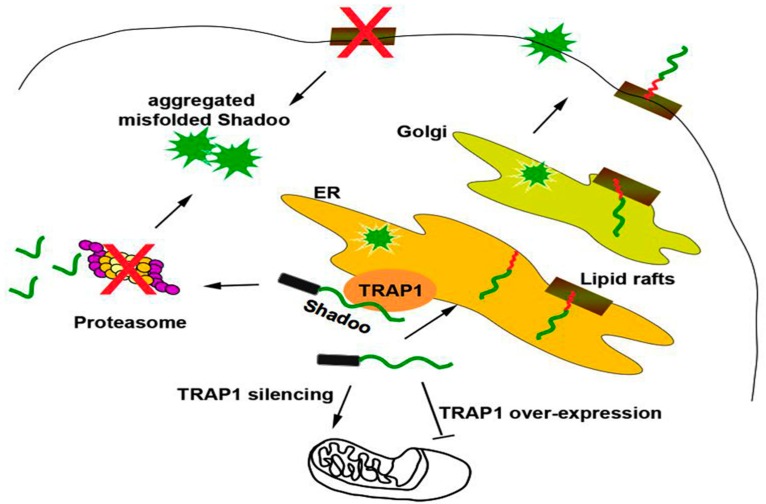
The molecular chaperone TRAP1 controls the dual ER/mitochondrial targeting of Shadoo. Shadoo is a secretory GPI-AP which is partially localized in the ER. In neuronal cells, Shadoo exhibits a strong tendency to misfold and contrary to canonical secretory proteins, it follows a dual targeting to ER or mitochondria regulated by the mitochondrial chaperone TRAP1 at the interface between ER/mitochondria. The folding properties of Shadoo are partially dependent on association to lipid rafts, whose alteration, as well as proteasomal block, induces its misfolding. Black rectangle: signal peptide; red “*zig-zag*” line: GPI-anchor. Arrows point to directionality of protein trafficking.

Intriguingly, we discovered that the IDP Shadoo was partially localized in the ER, where it interacted with the ER chaperone Calreticulin, exhibiting a strong tendency to misfold in neuronal cells, and, contrary to canonical secretory proteins, it followed a dual targeting to ER or mitochondria regulated by the mitochondrial chaperone TRAP1 at the interface between ER/mitochondria [22]. Furthermore, we showed that Shadoo possesses folding properties partially dependent on lipid rafts association, whose alteration, as well as proteasomal block, exacerbated its misfolding (Figure 4).

As Shadoo was found to control PrP structural dynamics and to increase prion pathological conversion [76], the role of misfolded Shadoo in the metabolism of PrP and its pathological mutants remains to be established.

Interestingly, recently, Ventura’s group [77] provided compelling experimental evidence for the presence of specific sequences, named “soft amyloid core” potentially able to induce the conformational conversion of prion-like domains in human nucleic acid binding proteins associated with diseases, such as microcephaly or cancer (DDX5, EYA1, ILF3, MED15, NCOA2, PHC1 and TIA1).

Aβ is a well known IDP with a wide range of oligomeric forms. Aβ monomers are able to polymerize producing soluble oligomers consisting of low-molecular weight aggregates, known to be the primary toxic agents responsible for neuronal dysfunction in AD. Larger and insoluble segments of Aβ precipitate as amyloid fibrils. However, the precise mechanism of the neurotoxic effects of Aβ peptides remains unclear.

Tau is an IDP whose primary function consists in maintaining the dynamic instability of neuronal microtubules. The conformational flexibility of tau covers a critical role in its own function [78]. In Alzheimer’s and other tauopathies, tau is able to form insoluble aggregates preceded by the presence of hyperphosphorylated tau monomers. Recent details on conformational shifts in the full-length IDP tau were given by the finding that the GSK-3β hyperphosphorylated tau monomers had an increasing tendency to form amyloids. In particular, hyperphosphorylation results in the development of new intramolecular interactions in the microtubules binding region and exposure of the amyloidogenic H2 region (tau hexapeptide ^306^VQIVYK^311^) [79].

Tau mutations linked to disease have been described to enhance binding of tau to soluble tubulin [80], as well as disrupt the interactions with microtubules [81] impacting on the stability of these latter. Indeed, tubulin-bound tau adopts an open conformation, characterized by diminished contacts between both N- and C- termini and the MTBR (microtubules binding region), thus defining a different conformational state respect to its aggregation-prone one, which exhibits a relatively compact ensemble [78]. Overall, the disordered nature of tau yields the flexibility to allow for conformational changes. In more detail, pathogenic tau adopts a β-pleated conformation [79] conferring high level of hydrophobicity and the ability to bind non-specifically to itself and other proteins [82], such as the case of association between pathological tau and ERAD proteins, giving rise to impairment of the ERAD functions [83].

## 5. Interactions between Misfolded Proteins and Plasma Membrane

The fibrillar assemblies derived from proteins associated with AD and prion disease differ by their surfaces because of diversity in their primary structures. These differences give rise to definition of the term “strains” for a given protein (such as the case of α-synuclein in synucleopathies) [84], which, as a consequence, has different interactomes and biophysical properties. Although the strain concept has been well documented for PrP^Sc^ [85], it is very new referred to Aβ and tau, for which, however, strain-dependent pathologies have been also reported [86,87].

### 5.1. Interaction with ECM Components

The distinct surface properties of pathogenic protein assemblies regulate the different affinities for molecules at the cell surface [10].

In this context, related to the prion-like mechanism of folding/misfolding and spreading, we will focus on the interaction of the specific misfolded protein assemblies, starting from their release from one cell to their binding to the cell surface of neighboring cell, without covering the intercellular transfer by tunnelling nanotubes [88] because this process circumvents the release and uptake routes.

Protein and lipid composition of the plasma membrane and extracellular matrix components (ECM) affect the interaction with misfolded proteins, thus contributing to their binding and further aggregation.

These observations were supported by experimental proofs by which, in knockout mice for any of the major component of the ECM, such as aggrecan (chondroitin sulfate proteoglycan) or tenascin-R (extracellular glycoprotein), the internalization of tau increased, as well as its spreading [89]. In addition, neurons with abundant ECM components did not show accumulation of NFTs in AD [90] and the secreted glycoprotein reelin was shown to exhibit protective neuronal activity against binding to Aβ, delaying fibril formation [91], thus leading to the postulation that ECM components may act as a barrier against misfolded assemblies at the plasma membrane.

In contrast, ECM components such as heparan sulfate proteoglycans (HSPGs) direct the binding and regulate the internalization of misfolded assemblies like PrP [92] and tau [93] or Aβ [94], for which HSPGs act promoting also the conversion of non-fibrillar Aβ into neurotoxic fibrillar forms in AD.

Thus, while chondroitin-sulfated proteoglycans may protect neurons from pathogenic proteins, heparan sulfate ones increase neuronal susceptibility to pathogenic attack.

Besides the already known indirect interaction mediated by HSPGs between PrP^C^ and non-integrin laminin receptor 37/67 kDa LR [95,96], we have recently found that PrP^C^ is able to directly bind 37/67 kDa LR in neuronal cells, and that a small organic naphtol-derived compound possesses the ability to control both their binding and their trafficking in neuronal cells, thus representing a new small molecule to be tested, at least against prion disease [97].

### 5.2. Interaction with Plasma Membrane

Besides ECM components, lipids and proteins of the plasma membrane are the main interactors of misfolded protein assemblies.

The Aβ aggregation process is stochastic and involves both homotypic (Aβ–Aβ) and heterotypic interactions (Aβ with other partners). Membrane lipids constitute important members of Aβ heterotypic interactors and have been described to modulate the generation of a wide variety of biochemically distinct oligomer sub-types [98].

Among lipids, cholesterol and the ganglioside GM1 are strong interactors and modulators of Aβ aggregation [99,100] and brain lipid rafts have the critical role to induce oligomerization and aggregation of Aβ by nucleating fibril formation [101]. Moreover, changes increasing the viscosity and order of lipid rafts in AD brains, respect to control ones, were determined at early stages of AD [102].

In addition, pathogenic protein assemblies are able to cluster on the plasma membrane of neuronal cells triggering deleterious events, such as changes in lipid bilayer architecture and fluidity, mediating neurodegeneration [10].

Amongst other alterations, lipid rafts from AD frontal cortex displayed low levels of n-3 long-chain polyunsaturated fatty acids and a general reduction of unsaturation and peroxidability indexes. These changes, together with the accumulation of β-secretase BACE1/APP in lipid rafts even at the earliest stages of AD, underline a connection between lipid alteration in lipid rafts and amyloidogenic processing of APP [103].

The involvement of lipid rafts in tau hyperphosphorylation has been described by Hernandez et al. [104]. They have suggested a role for cdk5 kinase (which, together with GSKβ, is known to control tau phosphorylation) and lipid rafts, in the early event of AD pathogenesis promoted by Aβ-induced signalling cascade in SHSY5Y neuroblastoma cells. Indeed, Aβ-induced Ser396/404 tau phosphorylation occurred in lipid rafts.

Because of the availability of pharmacological agents, among protein interactors of pathogenic assemblies, we can just mention the Aβ misfolded assemblies interaction with a PrP^C^-mGluR5 complex [105]. Indeed, knocking down of mGluR5s delayed neurodegeneration [106]. Membranous PrP^C^ has been identified as a ligand for the G protein coupled receptor Gpr126 in Schwann cells [107] and an interactor of aggregated PrP (e.g., PrP^RES^) [108]. Given that PrP^C^ is an entirely extracellular GPI-linked protein molecule, it is unclear how it might trigger lethal signal across the cell surface. However, PrP^C^ might mediate neurotoxic signaling by interacting, directly or indirectly, with plasma membrane molecules.

As mentioned in the introduction, the role of PrP association with lipid rafts in the folding and PrP^Sc^ formation has been studied in depth by us and others [4,5,109]. It emerges that, although both PrP^C^ and PrP^Sc^ are present in lipid rafts extracted from infected cells and from mouse brain [110], PrP^C^- and PrP^Sc^-associated rafts seem to have distinct characteristics, indicating either that the types of rafts associated with each isoform differ or that each isoform differ in the modality of association with lipid rafts (see [5] for details). However, the mechanism by which rafts can control misfolded PrP^Sc^ formation is still an open issue.

All together, these findings open new avenues for therapeutic development targeting the interactions of misfolded pathogenic proteins with the plasma membrane components.

## 6. Conclusions

The mechanism by which disease-associated proteins misfold, aggregate and form cellular toxic elements is an arduous issue that the active ongoing research is challenging. The examination of amyloid-forming proteins and their interactions with other molecular partners constitute the basics for identification of novel therapies against multiple disease states.

A considerable amount of research braces the conclusion that both Aβ and tau can aggregate and spread in the brain by a prion-like mechanism. The prion seeding model yields stringent evidence for a mechanism by which Aβ seeds act to support and perpetuate pathology of Alzheimer’s disease. Based upon in vitro and in vivo evidence, the formation of large protein aggregates in the cells has a protective role [111], so much so that the histopathological signatures of various neurodegenerative diseases, such as the amyloid plaques and NFTs in Alzheimer’s disease, the aggregated prions in prion diseases and the Lewy bodies in Parkinson disease, depict an attempt of the cells to shrink the damage caused by the small undesired oligomers [111].

Several pieces of evidence in literature so far indicate that cell chaperones, besides their well-known role in maintaining the proteins in their soluble native state, act to bind to protein oligomers and fibrils for neutralizing their effects (Figure 2) [112]. Indeed, the molecular chaperones can directly interact with the hydrophobic surfaces exposed by the oligomers/fibrils that can mediate aberrant interactions with various targets in the cells and on the plasma membrane. Moreover, the chaperones can be able to convert the oligomers into large and innocuous species by clearance mechanism, such as autophagy. Nevertheless, the mechanism of chaperone-induced in vivo formation of large aggregates and the benefits that human brains and tissues can obtain from this possible protective process remain an open issue that deserves further investigation.

## Abbreviations

AD	Alzheimer’s disease
ALS	amyotrophic lateral sclerosis
APP	amyloid precursor protein
Aβ	amyloid beta
BSE	bovine spongiform encephalopathy
CJD	Creutzfeldt–Jacob Disease
CTFβ	C-terminal fragment beta
ECM	extracellular matrix
ERAD	ER-associated degradation
FFI	Fatal Familial Insomnia
GPI	glycosyl-phosphatidyl-inositol
GSS	Gerstmann–Sträussler–Scheinker
HSPGs	heparan sulfate proteoglycans
IDD	intrinsically disordered domain
IDP	intrinsically disordered protein
MAPT	microtubules-associated tau protein
MTBR	microtubules binding region
NFTs	neurofibrillary tangles
PD	Parkinson’s disease
PrP^C^	cellular prion protein
PrP^Sc^	scrapie prion protein
RESET	ER stress-induced degradation
UPR	unfolding protein response
